# Ultra-Fast Middle-Up Reversed Phase Liquid Chromatography Analysis of Complex Bispecific Antibodies Obtained in Less Than One Minute

**DOI:** 10.3390/pharmaceutics14112315

**Published:** 2022-10-28

**Authors:** Amarande Murisier, Valentina D’Atri, Sebastian Pirner, Vincent Larraillet, Szabolcs Fekete, Matthew Lauber, Davy Guillarme

**Affiliations:** 1 School of Pharmaceutical Sciences, University of Geneva, CMU-Rue Michel Servet 1, 1211 Geneva, Switzerland; 2 Institute of Pharmaceutical Sciences of Western Switzerland, University of Geneva, CMU-Rue Michel Servet 1, 1211 Geneva, Switzerland; 3 Roche Diagnostics GmbH, Nonnenwald 2, 82377 Penzberg, Germany; 4 Waters Corporation, CMU-Rue Michel Servet 1, 1211 Geneva, Switzerland; 5 Waters Corporation, 34 Maple Street, Milford, MA 01757-3696, USA

**Keywords:** ultra-short columns, bispecific antibodies, reversed-phase liquid chromatography, improved selectivity, method robustness

## Abstract

This work illustrates the benefits and limitations of using ultra-short reversed phase liquid chromatography (RPLC) columns for the characterization of various complex bispecific antibodies after prolonged thermal stress at the middle-up level of analysis. First, we have demonstrated that alternative organic modifiers, such as isopropanol, can be used in RPLC mode without generating excessive pressure, thanks to the prototype 10 × 2.1 mm, 2.7 µm particle column. However, compared to acetonitrile, the selectivity was not improved, at least for the selected biopharmaceutical products. Importantly, very fast separations (sub-1 min) of high quality were systematically obtained for the different samples when using a spectroscopic detector, but a severe loss of performance was observed with mass spectrometry (MS) detection due to dispersion effects. Based on these results, there is a clear need to improve the interfacing between LC and MS (shorter/thinner tubing) to mitigate band broadening.

## 1. Introduction

Antibody-based products are continuously growing in structural complexity and therapeutic applications [1]. Among them, bispecific antibodies (bsAbs) certainly hold a prominent place because of their customizable structure that is able to recognize multiple antigen sites. These new antibody-based formats have made clear the fact that new analytical needs are required in the field of therapeutic proteins [2,3,4]. Accurate structural characterization and high throughput analyses are the new buzzwords for biopharmaceutical companies that are facing an era of extremely crowded pipelines and clinical trials [2]. Fortunately, advances in therapeutic protein formats go hand in hand with advances in analytical and separation science like the innovative technologies that have been proposed to achieve fast separations in LC, namely columns packed with sub-2 µm totally porous and sub-3 µm superficially porous, pore-size optimized particles [5]. Indeed, due to the ability of large molecules (as therapeutic proteins) to experience an “on-off” elution mechanism in gradient separations, it has been proved that column length has a minor impact on their separations [6]. This concept led to the idea of further reducing the length of chromatography columns, and more recently, it has been demonstrated that ultra-short columns (5 or 10 mm length) might result in comparable separation quality in comparison to standard columns having lengths of 50, 100, or 150 mm when analyzing large solute such as therapeutic proteins [7,8]. The clear advantages of using ultra-short columns lie in obtaining very rapid analyses or using alternative organic solvents (such as isopropanol) while running instrumentation and subjecting samples to lower operating pressures.

In the present work, a prototype RPLC ultra-short column (particle size 2.7 µm, column dimensions 10 × 2.1 mm, pore size 450 Å) was used to analyze complex bsAbs at the middle-up level to monitor modifications occurring after prolonged thermal stress. For the first time, a liquid chromatography-fluorescence-mass spectrometry (LC-FLR-MS) analysis of bsAbs subunits was performed in less than 1 min and that was made possible through the use of ultra-short columns. The advantages and limitations of this new experimental approach are discussed in detail.

## 2. Materials and Methods

### 2.1. Chemical and Reagents

Type 1 water was obtained by a Milli-Q purification system from Millipore (Bedford, MA, USA). LC-MS grade acetonitrile (ACN) and isopropanol (IPA) were purchased from Fisher Scientific (Reinach, Switzerland). MS-grade difluoroacetic acid (DFA) (Ionhance DFA) was obtained from Waters (Milford, MA, USA). DL-dithiothreitol (DTT) (for molecular biology, ≥98% HPLC), guanidine hydrochloride (for molecular biology, ≥99%), TRIZMA base (≥99.8%), and hydrochloric acid (HCl) solution (1.0 N, BioReagent) were purchased from Sigma-Aldrich (Buchs, Switzerland). Bispecific antibody (bsAb)-related complex samples including mAb-domain-fusion (C-terminal) protein sample (∼200 kDa), mAb-cytokine-fusion protein sample (∼165 kDa), mAb-domain-fusion (N-terminal) protein sample (∼200 kDa) and Fab domain (∼50 kDa) were provided by Roche (Penzberg, Germany) and referred as bsAb1, bsAb2, bsAb3, and bsAb4, respectively. Thermally stressed samples were obtained by incubation at 40 °C for several weeks.

### 2.2. Apparatus and Methodology

#### 2.2.1. RPLC Analyses for Organic Modifiers Comparison

RPLC analyses were performed using 0.05% (*v*/*v*) DFA in water as mobile phase A, and 0.05% (*v*/*v*) DFA in organic modifier as mobile phase B. 100% ACN, 100% IPA and 50/50 ACN/IPA (*v*/*v*) in mobile phase B were compared. Experiments were performed on a Waters Acquity I-Class system, equipped with a binary solvent delivery pump, an autosampler, a fluorescence (FL) detector (2 μL FL flow cell), and a flow-through needle (FTN) injection system with a 15 μL needle. In the present work, FL was used instead of UV, to maximize sensitivity and allow the visualization of minor species. It is however important to notice that on our instrument, the FL flow cell has a lower upper-pressure limit (35 bar) vs. the UV cell (70 bar). FL-chromatograms were acquired at λ_excitation_ = 280 nm and λ_emission_ = 350 nm. The flow rate was set at 400 µL/min and the gradient time at 2 min. 0.5 µL volumes of each sample were injected in each condition. When using ACN as an organic modifier, linear gradients were run from 30 to 42%, 28% to 56%, and 28% to 40% of mobile phase B, for bsAb1, bsAb2, and bsAb3, respectively. When using ACN/IPA as an organic modifier, linear gradients were realized from 28 to 40%, 26% to 54%, and 27% to 39% of mobile phase B, for bsAb1, bsAb2 and bsAb3, respectively. When using IPA in mobile phase B, linear gradients were realized from 24 to 36%, 22% to 50%, and 22% to 34% of mobile phase B, for bsAb1, bsAb2, and bsAb3, respectively. Data acquisition and instrument control were performed by Empower Pro 3 software (Waters). 

#### 2.2.2. RPLC-MS Analyses

RPLC-MS experiments were performed on an Acquity UPLC I-Class PLUS system, coupled to an FL detector (λ_excitation_ = 280 nm and λ_emission_ = 350 nm) and a high-resolution BioAccord ToF mass spectrometer from Waters. A prototype BioResolve RP mAb Polyphenyl column (2.7 μm, 10 × 2.1 mm, 450 Å) from Waters was used. The column temperature was set at 70 °C. Mobile phase A was 0.05% (*v*/*v*) DFA in water, and mobile phase B was 0.05% (*v*/*v*) DFA in ACN. The injection volume was 0.5 µL. Optimized linear gradients were run in 1 min (without re-equilibration) from 30% to 36%, 26% to 54%, 29% to 37% and 25% to 38% of mobile phase B, for bsAb1, bsAb2, bsAb3 and bsAb4, respectively. The LC flow rate was set at 1000 µL/min and split with a PEEK T-junction so that the flow rate entering the MS was equal to 275 µL/min. The MS was used in ESI-positive mode with an acquisition range of 400 to 7000 m/z. A 200 pg/µL sodium iodide solution diluted in a mixture of 50/50 water/IPA (*v*/*v*) with 0.1% formic acid was used as a mass spectrometer calibrant. The desolvation temperature was set at 550 °C, the source temperature at 120 °C, the cone voltage at 30 V, and the capillary voltage at 1.5 kV. Data acquisition and instrument control were performed with Unifi software (Waters, Milford, MA, USA) and mass spectra data treatment was performed with MassLynx software (Waters, Milford, MA, USA).

### 2.3. Sample Preparation

**Figure 1:**
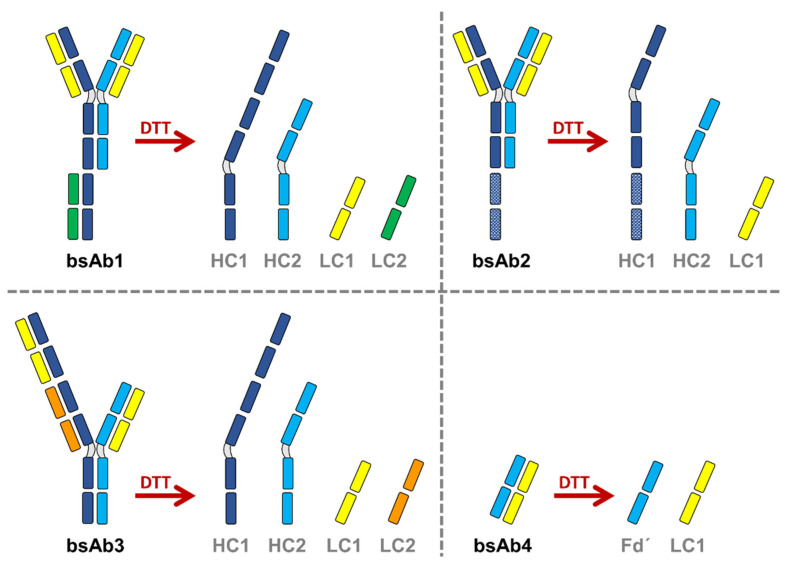
Schematics of the different bispecific antibodies (bsAbs) with the corresponding subunits resulting from the chemical reduction performed with DL-dithiothreitol (DTT). HC and LC stand for heavy chain and light chain, respectively.

Samples were first reduced in a denaturing buffer (0.4 M Tris, 8.0 M GuHCl, pH 8.0), prepared by dissolving 4.85 g Tris base and 76.42 g guanidinium hydrochloride in 100 mL of water. The pH value was adjusted to 8 using a solution of 25% HCl. DTT was added in the denaturing buffer to reach a final concentration of 0.04 M. This solution, prepared fresh before use, was added to 2 mg/mL solution of bsAb in water (1:1) to obtain a final concentration of bsAb of 1 mg/mL. The DTT antibody solution was then incubated for 30 min at 37 °C. The prepared samples were analyzed within 2–3 days, provided that they were stored at 4 °C. Depending on the initial bsAb product undergoing reduction, different protein subunits were generated, as illustrated in Figure 1.

## 3. Results and Discussion

### 3.1. Evaluation of Alternative Mobile Phase Eluents

In the RPLC of proteins, acetonitrile remains the most widely used mobile phase component. However, as already demonstrated elsewhere [9], the chromatographic selectivity can possibly be altered by changing the nature of the organic modifier. This behavior could be attributed either to the modification of weak chemical interaction (acetonitrile is aprotic, while alcohol-based modifiers are protic, so H-bond interaction may be strongly modified) or to the fact that mobile phase viscosity could vary significantly when changing the nature of the organic solvent. Indeed, it has been recently shown that the selectivity of proteins can be significantly modified with pressure changes [10]. A change from acetonitrile to an alcoholic mobile phase system might also bring to effect π-π electron interactions between a phenyl-bonded stationary phase and protein amino acid side chains, such as tyrosine, tryptophan, and phenylalanine [10].

**Figure 2:**
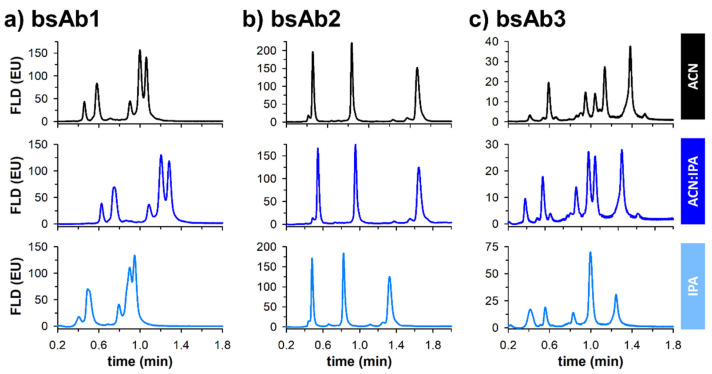
RPLC-Fluorescence chromatograms of the different bispecific antibodies (bsAbs), namely bsAb1 (**a**), bsAb2 (**b**), and bsAb3 (**c**), by using 3 different organic modifiers as mobile phase B, namely acetonitrile (ACN, black), 50:50 acetonitrile:isopropanol (ACN:IPA, blue), and isopropanol (IPA, cyan). Difluoroacetic acid (DFA) was used as the acid modifier in all mobile phases at a concentration of 0.05% (*v*/*v*).

In the present work, ultra-short columns were employed, generating reasonable backpressure. Therefore, it becomes possible to use isopropanol (IPA), which is known to be highly viscous, as an eluent instead of acetonitrile, without instrumental limitations. Besides pure IPA, a mixture of 50:50 IPA:ACN was also tested in this work. In each case, these mobile phases were acidified with 0.05% of difluoroacetic acid (DFA). Figure 2 shows the corresponding fast separations obtained for three reduced bispecific mAb products using these three different types of mobile phases. First, it is important to note that pressure drop remained acceptable whatever the conditions. Indeed, ΔP was equal to 110, 160, and 280 bar when using ACN, ACN:IPA, and IPA as the eluent, respectively. However, even if the pressure remained within acceptable limits, the added value of IPA was found to be limited in the three selected examples.

Indeed, the separation performance was highly comparable for bsAb1 when using pure ACN or the ACN:IPA 50:50 mixture, but the overall performance was strongly reduced with pure IPA, with a severe loss of selectivity between the most critical peak pair, and a reduction of retention. For the second example (bsAb2), selectivity between the three major species and the minor variants remains identical whatever the mobile phase components, and only the retention was reduced with IPA. Finally, for the third example (bsAb3), the selectivity was significantly modified depending on the nature of the eluent. The separation carried out with IPA was clearly the worst one, with a loss of selectivity between two major peaks. Even if no selectivity improvement was noticed with IPA for the selected examples, Figure 2 proves that selectivity can be tuned by modifying the solvent composition of the eluent. Interestingly, the use of ultra-short columns for protein analysis under RPLC conditions provides more flexibility in terms of organic solvent because it is unlikely to run into the pressure limits of the LC instrumentation.

### 3.2. Ultra-Fast Separations of Complex Mab Products

**Figure 3:**
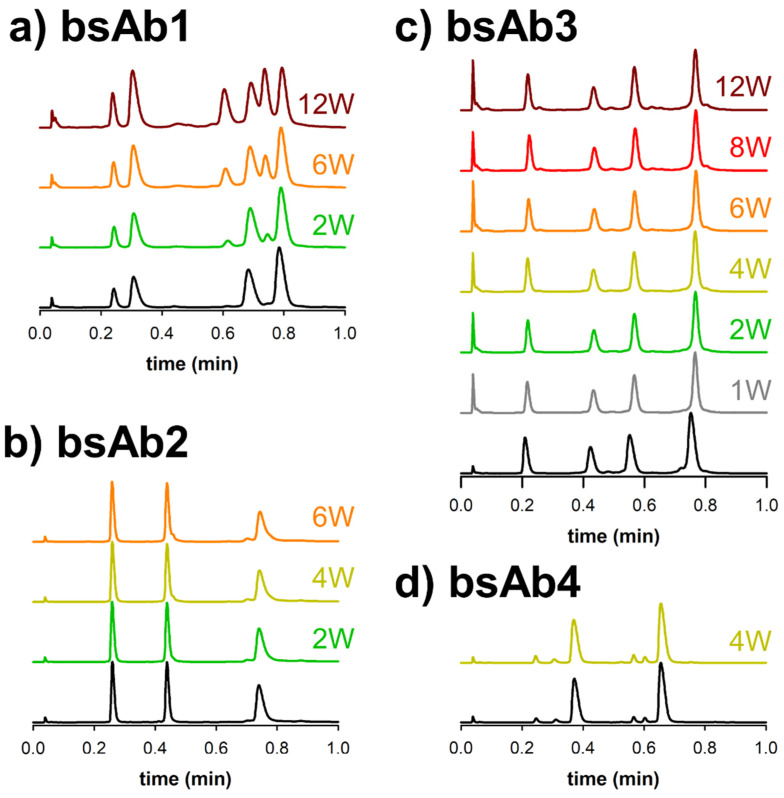
RPLC-Fluorescence chromatograms of the different bispecific antibodies (bsAbs), namely bsAb1 (**a**), bsAb2 (**b**), bsAb3 (**c**), and bsAb4 (**d**) acquired before (black) and after a thermal stress performed at 40 °C for 1 (grey), 2 (green), 4 (lime), 6 (orange), 8 (red), or 12 (brown) weeks.

A clear benefit of using ultra-short columns for the analysis of therapeutic proteins is the possibility to reduce analysis time, with limited impact on overall kinetic performance. Such a behavior is demonstrated in Figure 3. 

Various sub-1 min separations of complex biopharmaceutical samples are displayed as detected by native protein fluorescence. In this case, both the reference samples and various thermally stressed samples (from 1 to 12 weeks at 40 °C) were analyzed. Only the samples reduced with DTT were analyzed (sub-units described in Figure 1), as the chromatograms were found to be much more informative than the ones obtained at the intact level. For comparison purpose, the same samples were already analyzed using multi-isocratic elution mode [11]. To maximize separation power, the composition ranges were adjusted depending on the product hydrophobicity, while the gradient time and flow rate were constant, 1 min and 1 mL/min, respectively. This ensured to have the highest possible k* value (k* corresponds to the gradient retention factor which has a similar significance in gradient elution as the retention factor k in isocratic mode). In the present case, k* values ranged from 0.72 to 3.38 for the four different samples. In addition, no equilibration time was added at the end of the run, since the volume of the 10 × 2.1 mm I.D. column was equal to about 21 µL, corresponding to a column dead time of only 1.3 s when working at a flow rate of 1 mL/min. So, the injection time (about 30 s) was already sufficient to equilibrate the column between each run, as it corresponds to more than 20 column volumes. It is also important to mention that all system dispersion effects were reduced as much as possible to limit band broadening. To do this, we limited the injection volume to only 0.5 µL, used 220 mm × 65 µm tubing between the column outlet and detector inlet, and employed a commercial 2 µL fluorescence flow cell. Despite these extra considerations and taking into account the k* value, performance achieved on the 10 × 2.1 mm column was slightly lower than expected, and additional band broadening was observed due to the presence of both extra-column volume and extra-bed volume. [8] 

Figure 3a shows the ultra-fast separation of bsAb1 before and after a thermal stress of 2, 6 and 12 weeks. As expected, four main species were observed on the reference chromatogram (no stress), with the first two peaks corresponding to the two different light chains (LC1 and LC2), and the last two peaks corresponding to the two heavy chains (HC1 and HC2). After the thermal stress, two additional major peaks were observed in the elution region corresponding to the HC, and a minor species was observed between the elution zones of the LC and HC species. These extra species were already present in the reference sample, but in very low amounts (<1%). The chromatograms reported in Figure 3a clearly show that the amount of the two main impurities was increasing significantly with stress time, and this amount can be easily quantified with the fluorescence detector, based on peak areas (yet this is out of the scope of the present work). 

Similar conclusions were obtained for the three other biopharmaceutical products (i.e. bsAb2, bsAb3 and bsAb4). For bsAb2, LC1 and HC2 were seen to closely elute as the first and second peaks, while HC1 was more hydrophobic and eluted later. Here again, two additional species were observed on the stressed sample, including one that eluted just after HC2 and another that eluted before HC1. The amount of impurities related to the degradation from thermal stress (degradation related impurities) increased very slightly with stress time, even after 6 weeks at 40 °C. For bsAb3, four species were expected according to Figure 1. Based on their hydrophobicity, the two LC species would be predicted to elute first, and before the molecule’s two HC subunits. Here again, we observed some differences in terms of minor species. Several various additional species were introduced to the sample over time and found to elute between the main species. Nevertheless, the amount of impurities was found to be limited even after 12 weeks of thermal stress. For bsAb4, only two samples were available, namely the reference sample and the sample exposed at 40 °C for 4 weeks. The subunit species predicted for this molecule is shown in Figure 1. In the chromatogram, only two main species were observed: LC1 eluted as the first peak, while Fd’ eluted as the second. Four additional species were already observed in minor quantities and separated on the reference sample. Interestingly, the amount of these four impurities did not increase through thermal stress.

As illustrated in this section, ultra-fast analytical methods are very helpful for rapidly checking a sample for additional variants that are induced by stress, in biopharmaceutical samples over time.

### 3.3. Coupling Ultra-Fast Separations with MS

**Figure 4:**
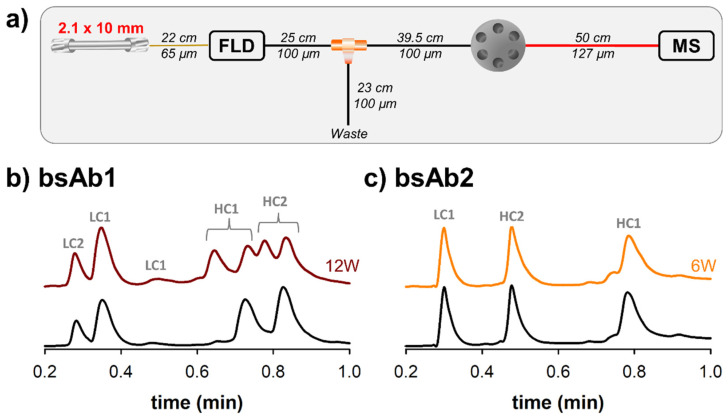
RPLC-Fluorescence-MS analysis of bsAbs. (**a**) Schematic representation of the instrument configuration reporting the length and the internal diameter of the tubing used to realize the liquid chromatography-Fluorescence-Mass Spectrometer (LC-FLD-MS) flow path and the flow splitting. Total ion chromatograms (TICs) of the different bispecific antibodies (bsAbs), namely bsAb1 (**b**) and bsAb2 (**c**) acquired before (black) and after thermal stress performed at 40 °C for 6 (orange) or 12 (brown) weeks. HC and LC stand for heavy chain and light chain, respectively.

In order to obtain more information on the peaks observed in Figure 3, we have also performed some experiments with ultra-short columns using both fluorescence (quantitative detector) and MS (qualitative detector) detectors. The experimental setup employed for this part of the work has been described in Figure 4a. Next to the fluorescence detector, a flow splitter was added to provide an optimal flow rate to the ESI-MS source. A flow rate of 275 µL/min was split to the MS detector, while the remaining flow (725 µL/min) was directed to waste. In addition, it was mandatory to add a switching valve before the ESI inlet, allowing a calibrant solution to be periodically infused for real-time lock mass adjustment. In the end, based on the minimal distances between the different parts of the MS device, three tubes of 250 mm × 100 µm, 395 mm × 100 µm and 500 mm × 127 µm had to be added between the fluorescence detector and ESI-MS source. It has already been demonstrated elsewhere that the tubes located between the ultra high performance liquid chromatography (UHPLC) system and the ionization source are particularly critical for minimizing band broadening [12]. In the present case, the added tubing corresponds to a total volume of more than 11 µL, so band broadening may be quite severe when using ultra-short column with a volume of only 21 µL. Unfortunately, thinner tubes cannot be used to reduce the volume, as the fluorescence flow cell has an upper pressure limit of only 35 bar and thinner tubes would generate a pressure higher than this cut-off value. As an alternative, UV detection (the UV cell has a higher upper pressure limit of 70 bar, for the instrument used in this work) could be envisaged to provide more flexibility to the instrumental setup, but sensitivity would then be reduced.

Total ion chromatograms (TICs) were recorded for all four samples using the conditions described in Section 3.2, but with the addition of MS detection. Only the traces corresponding to the unstressed and stressed bsAb1 and bsAb2 samples were reported in Figure 4. A similar behavior was observed for bsAb3 and bsAb4.

The two chromatograms reported in Figure 4b should be compared with the ones reported in Figure 3a using the same color code. As expected, the retention and selectivity were comparable between these two separations, as only the detector changed. However, the peaks were much broader when using MS detection (at least 2-fold broader), leading to a non-negligible loss of resolution. To better understand the reason for this loss in performance, we have tried to modify the MS settings. Various scan rates ranging from 2 to 10 Hz were tested, but unfortunately, only sensitivity was altered with the higher data acquisition rate, while the peak broadening remained comparable. Next, we also tried to bypass the fluorescence detector, but here again, the impact on final separation was minor (less than 10% improvement in peak widths). Based on these observations, it is clear that the severe band broadening observed with MS detection was almost exclusively due to the tubing added between the fluorescence detector outlet and ESI-MS inlet. It is indeed important to keep in mind that extra column band broadening in a tube (σ^2^_tubing_) is inversely proportional to the diffusion coefficient of the analyzed molecule (D_m_), as reported elsewhere [13]. In the present case, the analyzed species have sizes varying between 25 and 75 kDa, corresponding to calculated diffusion coefficients (using the Wilke-Chang equation, which is known to be quite precise [14]) comprised between 4.1 and 8 × 10^−11^ m²/s under the mobile phase conditions employed in this work. These values are in average 10 to 20-fold higher than the standard value considered for small molecules (D_m_ = 1 × 10^−9^ m^2^/s), thus explaining the significant contribution of the tubing to band broadening. 

Similar chromatography was observed in Figure 4c, which presents LC-MS chromatograms of bsAb2. These separations should be compared with the ones reported in Figure 3b. In this example, the loss in performance due to MS was even more severe than for bsAb1, since a 3-fold loss in peak capacity was noticed. One possible explanation is related to the gradient span. Indeed, bsAb1 and bsAb2 were analyzed with a gradient from 30 to 36% B, and from 26 to 54% B, respectively. Therefore, the k* values were almost 5-times lower for bsAb2 vs. bsAb1 analysis (k* was equal to 0.72 for bsAb2 and 3.38 for bsAb1), thus reducing the column variance and increasing the impact of extra-column variance (it remains comparable between the two examples) on peak broadening.

In the end, an interesting solution to limit band broadening with MS detection would be to discard the fluorescence detector from the experimental setup or to have it plumbed against a splitter so that it can receive column effluent in parallel with the MS detector. In either case, thinner tubing could be employed prior to the ESI-MS inlet, without the risk to destroy the fluorescence flow cell. Special considerations would need to be made with the ToF instrument employed here, because it uses a fused silica transfer line that is directly integrated and crimped onto the electrospray capillary of the mass spectrometer.

## 4. Conclusions

This work clearly highlights the benefits, but also the limitations, of using ultra-short columns for the characterization of complex biopharmaceutical products. 

Even if selectivity was not improved with IPA vs. ACN as an RPLC eluent with the selected bispecific antibodies, it is important to keep in mind that ultra-short columns allow the use of more viscous mobile phases because of their inordinately high permeability and low operating pressures.

In effect, this work shows that it is possible to perform highly efficient sub-1 min analyses on biopharmaceutical products, more specifically relatively complicated bispecific antibody species that produce a mixture of analytes during a subunit level analysis. All the separations obtained with a spectroscopic detector were of high quality, but a loss in performance was observed with MS detection. For successful operation with MS detection, ultrashort columns will need to be coupled to mass spectrometers with more specially designed integration. The geometry of the tubing located between the column outlet and MS inlet should be carefully optimized. More ideally, the column should be made to operate as close as physically possible near the ESI probe. The success of a source-integrated, vacuum jacketed columns shows that new engineering considerations for this pursuit are now at hand [15,16]. The optimization of ultrafast protein separations for improved MS hyphenation will bring a great deal of high throughput, detail rich information to biopharmaceutical developers and is thus a worthy endeavor.

## Data Availability

Not applicable.
